# Diagnostic Assessment of Maxillary Sinus Membrane Thickening Associated with Dental Implant Perforation Using Cone-Beam Computed Tomography: A Retrospective Cross-Sectional Pilot Study

**DOI:** 10.3390/diagnostics15212809

**Published:** 2025-11-06

**Authors:** Narjesse Kemcha, María Andrés-Veiga, Dolores Hurtado-Celotti, Cristina Meniz-García, Tomás Beca-Campoy, Natalia Martínez-Rodríguez

**Affiliations:** 1 Department of Dental Clinical Specialties, Faculty of Dentistry, Complutense University of Madrid, 28040 Madrid, Spain; nkmcha@ucm.es (N.K.); dhcelotti@ucm.es (D.H.-C.); nataliamartinez@ucm.es (N.M.-R.); 2 Faculty of Health Sciences, Alfonso X University (UAX), 28691 Madrid, Spain; mandrvei@uax.es; 3 Surgical and Implant Therapies in the Oral Cavity Research Group, Faculty of Dentistry, Complutense University of Madrid, 28040 Madrid, Spain; 4 Department of Surgery, Faculty of Medicine, University of Salamanca, 37008 Salamanca, Spain; tomasbc@usal.es

**Keywords:** maxillary sinus, dental implants, sinus floor perforation, sinus membrane thickening, CBCT

## Abstract

**Background/Objectives**: Perforation of the maxillary sinus floor by dental implants is a complication that can occur during treatment in posterior sectors; however, its clinical and radiological consequences remain controversial. Therefore, this study aimed to use the diagnostic value of CBCT to determine the possible association between sinus floor-perforating implants and their clinical and/or radiological impact. **Methods**: A retrospective observational study was conducted on CBCT scans from 21 patients with implants protruding into the maxillary sinus. Morphometric analysis was performed to assess sinus membrane thickening (SMT) patterns, height, surface area and density and the presence of sinonasal symptoms. Statistical analysis was used to explore potential associations between SMT and variables such as implant protrusion length, number of perforating implants, age and gender. **Results**: SMT was observed in all patients, with a mean area of 11.1 ± 6.4 mm^2^ in panoramic sections. Most cases (85.7%) exhibited a circumferential SMT pattern. No statistically significant correlation was found between SMT and implant protrusion length, age or sex. Additionally, none of the patients reported sinonasal symptoms, and no clinical signs of sinusitis were detected during follow-up. **Conclusions**: Although sinus floor-perforating implants commonly induce SMT detectable on CBCT, this thickening appears largely asymptomatic and may not compromise patient well-being in the short term. Nevertheless, clinicians should monitor these cases radiographically to detect possible long-term complications.

## 1. Introduction

The maxillary sinus is a pneumatic cavity located within the maxillary bone, whose main functions are to lighten the skull and contribute to voice resonance. It exhibits considerable anatomical variability in terms of size, shape, and its relationship with the dental roots. Among the most relevant variants is the course of the alveolar-antral artery, which runs along the lateral wall of the sinus and may adopt intraosseous, submucosal, or mixed pathways. Similarly, the presence of bony septa, known as Underwood’s septa, can partially or completely divide the sinus cavity, thereby complicating surgical procedures. These anatomical variations increase the risk of sinus membrane perforation during interventions such as maxillary sinus lift surgery [1].

Maxillary sinus involvement secondary to dental pathology or implant-related surgical interventions is being increasingly identified, facilitated by the routine integration of Cone-Beam Computed Tomography (CBCT) into diagnostic protocols [2].

Implant-supported rehabilitation remains the standard approach for the replacement of edentulous spaces [3,4]. From a clinical standpoint, two common presentations are observed: a deficiency in alveolar bone height or the presence of sufficient residual bone volume. In cases of vertical bone deficiency, maxillary sinus floor elevation procedures are frequently indicated. As described by Molina et al. [5], these interventions provide high predictability; however, they are not devoid of risk and may be associated with complications such as chronic rhinosinusitis, Schneiderian membrane perforation, implant migration and mucosal thickening.

Alternatively, when residual alveolar bone is insufficient, standard implant placement is generally uneventful. Nonetheless, sinus floor perforation may still occur, either inadvertently or deliberately to achieve bicortical engagement, the consequences of which for sinus mucosal integrity and function remain insufficiently characterised.

In one of the earliest investigations into the implications of sinus floor perforation, Nooh [6] deliberately extended implants 3 mm beyond the sinus floor. The findings indicated no adverse effects on implant stability or survival at the one-year follow-up. Subsequent studies investigating the consequences of sinus floor perforation have consistently demonstrated high implant survival rates while downplaying concerns regarding its effect on maxillary sinus health [7,8,9].

Conversely, authors such as Chaves et al. [10], in their study, and Ragucci et al. [11], in their systematic review, agree that sinus floor perforation during implant placement leads to sinus membrane thickening (SMT). Oliveira-Santos et al. [12] analysed 129 paranasal sinuses housing 202 dental implants, identifying sinus floor perforation in 46.5% of cases. This finding correlated with thickening of the sinus mucosa, which in some instances culminated in total opacification of their lumina. Brandstaetter et al. [13], from a substantial dataset of CBCT scans, identified a strong correlation between clinical maxillary sinus pathology and the presence of protrusive dental implants.

The disparities between existing studies underscore the need for further research to determine whether sinus floor perforation during implant placement poses a clinically significant risk. Consequently, the present study based on the diagnostic capacity of CBCT aims to evaluate the association between perforating implants and sinus mucosa thickening, with special emphasis on identifying clinical correlations, morphological patterns, perforation height and the degree of sinus opacification.

## 2. Materials and Methods

### 2.1. Study Design

A retrospective observational study was designed as per the recommendations of the STROBE initiative statement [14], was conducted in accordance with the Declaration of Helsinki’s ethical provisions and was approved by the Ethics Committee of the Hospital Clínico San Carlos de Madrid (CI 22/190-E, approved on 16 March 2022).

### 2.2. Context and Participants

A total of 264 patients were treated in the Implantology Service of the Faculty of Dentistry of the Complutense University of Madrid for implant placement in the posterior maxillary sectors, between August 2020 and January 2022.

In the planning CBCT scans, no pre-existing abnormalities of the maxillary sinus were detected in 121 of the 264 patients (45.83%). After one year, two researchers (N.K. and D.H.-C) reviewed the control CBCT scans, observing that 21 patients, selected according to the inclusion and exclusion criteria shown in Table 1, had sinus floor perforation.

### 2.3. Clinical Data Collection

The 21 patients chosen were called for clinical review and questioned about the appearance of any symptoms since the placement of the implant, such as facial pain, unilateral rhinorrhoea, hyposmia, nasal obstruction or cacosmia.

### 2.4. Radiological Data Assessment

The following radiographic data were extracted from the CBCT images: number of implants per sinus, assessment of lesion extent according to the treated side and the length in millimetres of the apical portion of the perforating implant perceived on the panoramic and orthoradial sections.

Membrane thickening assessment: this consisted in determining whether the SMT adopted a horizontal or circumferential pattern in both the panoramic and orthoradial sections and defining its height in millimetres.

To assess the radiographic changes between the initial scan (T0), which showed no sinus alterations, and the control scan (T1), where sinus membrane thickening appeared associated with implant perforation through the sinus floor, the membrane thickness was measured on both scans. Measurements were performed on standard panoramic and orthoradial CBCT sections, selecting the area of greatest thickening at T1 and the anatomically corresponding area at T0. The NNT Viewer software (version 10.0), provided by NewTom Imaging CBCT systems, was used for the measurements, calibrated in millimetres using the integrated measurement function. The delta was defined as the linear difference between sinus membrane thickness at T1 and the baseline value at T0: Thickness (T1) − Thickness (T0). Two independent examiners performed the measurements, and interobserver agreement was calculated using the intraclass correlation coefficient (ICC). In cases where discrepancies greater than 0.2 mm occurred, a joint consensus was reached.

The measured heights were used to classify SMT as follows: (1) Normal < 2 mm; (2) Mild-to-moderate > 2 mm; (3) Severe > 10 mm; (4) Total opacification, according to the classification proposed by Maillet et al. [15]. The latter suggests that subtypes 2, 3 and 4 should be considered as pathological.

**Figure 1:**
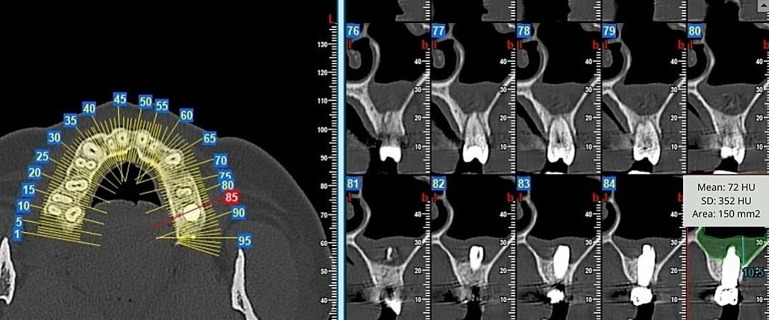
Measure of sinus membrane thickening in squared millimetres and Hounsfield Units on the orthoradial section.

**Figure 2:**
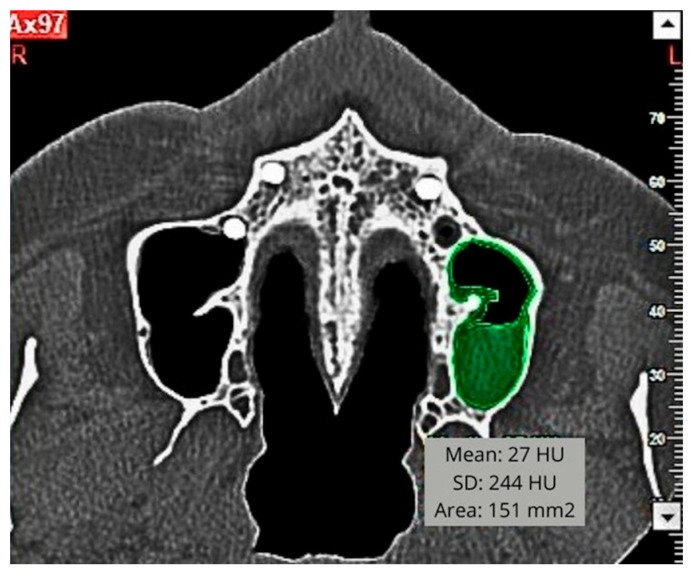
Measure of sinus membrane thickening in squared millimetres and Hounsfield Units on the axial section.

In the same scope, the thickened surface was measured in square millimetres, in the panoramic, orthoradial and axial sections; and its density was quantified in Hounsfield Units (HU) in the same sections of the CBCT scans (Figure 1 and Figure 2).

### 2.5. Morphometric Measurements

The dependent quantitative variables analysed were measured using the software NNT Viewer (version 10.0), normally used for 3D modelling and segmentation of computed tomographies, provided with the CBCT device NewTom Imaging (Bologna, Italy), with an FOV of 16 cm × 18 cm. In the panoramic section, a thickness of 10 mm was used, with a distance between slides of 1 mm. In the orthoradial section, a thickness of 0.3 mm was used.

These measurements were made by two researchers (N.M.-R., and M.A.-V.), recording a Kappa agreement coefficient of 0.8.

### 2.6. Statistical Analysis

The statistical program IBM SPSS Statistics for Windows (Version 27.0, IBM Corp., Armonk, NY, USA) was used to obtain a detailed description of the derived data with frequencies and percentages.

The relationship between the independent and main dependent variables was analysed using the Pearson correlation, and the Chi-square test was used to analyse the outcomes of crossing the qualitative variables with each other. Furthermore, a Student’s *T* test was performed to assess the associations of some qualitative variables with other quantitative variables. The level of significance was set at *p* < 0.05, and all confidence intervals were given at the level of 95%.

## 3. Results

A total of 21 patients, 11 women and 10 men, presented asymptomatic SMT, with a mean age of 70.3 ± 13.4 years, within a range of between 43 and 88 years.

### 3.1. Clinical Data Analysis

None of the symptoms suggesting sinusitis development in the 21 study patients were registered.

### 3.2. Radiological Data Analysis

The results, shown in Table 2, indicate that 61.9% of the patients (*n* = 13) had one perforating implant per sinus, whereas the remaining 38.1% (*n* = 8) had two perforating implants per sinus. Regarding the affected side, 11 patients (52.4%) presented SMT on the right maxillary sinus, whereas it was observed on the left maxillary sinus in 10 patients (47.6%).

The length of the protruding portions of the perforating implants in the sinus cavity, measured in millimetres, was comparable in both the panoramic (2.4 ± 1.5 mm) and orthoradial sections (2.3 ± 1.5 mm). The women presented slightly higher values of SMT in the orthoradial sections (9.31 ± 6.02 mm^2^) compared to male patients, who registered a mean of 8.25 ± 7.34 mm^2^, with no statistically significant differences (*p* = 0.236). There was no significant correlation with age (*p* = 0.96) or with the length of the perforating implants (*p* = 0.63).

As shown in Table 3, a circumferential thickening pattern was predominant, evidenced in 15 patients (71.43%), while the horizontal pattern was recorded in 6 patients (28.57%). There was no significant correlation (*p* = 0.531) between the number of perforating implants and SMT patterns, nor between the length of the protruding portion of the implants (*p* = 0.260).

The values of SMT area varied in the different sections. In the panoramic one, the SMT was between 78.84 mm^2^ and 506.34 mm^2^ (mean: 219.2 ± 107.7), whereas in the orthoradial section it ranged between 17.55 mm^2^ and 209.88 mm^2^ (mean: 100.8 ± 55.8). In the axial section, the registered values fluctuated from 60.12 mm^2^ to 404.37 mm^2^ (mean: 204.1 ± 99.7). Neither age (*p* = 0.18) nor the length of the perforating implants significantly affected the extent of SMT area.

The mineral density of SMT, expressed in HU, varied considerably between the sections. In the panoramic section they were 21.8 to 366.2 HU (mean 164.2 ± 97.8 HU); in the orthoradial section 35.8 to 703.4 HU (mean 222.5 ± 175.0 HU), and in the axial section 165.0 to 584.0 HU (mean 333.4 ± 131.5 HU). No statistically significant correlations were found.

The highest SMT values (T1-T0) recorded in the vertical measurements of the panoramic sections ranged from 2.4 mm to 21.3 mm (mean: 11.1 ± 6.4 mm). In the orthoradial sections, the values ranged from 2.1 mm to 23.6 mm (mean: 8.8 ± 6.5 mm), with a statistically significant difference between means (*p* = 0.008). According to the classification of Maillet et al. [14], the SMT values in these sections correspond to categories (2) and (3) and are thereby considered pathological.

For the purposes of assessing whether SMT values were gender-related, women had a mean of 12.75 ± 5.85 mm in the panoramic section, compared to men, who had a mean of 9.32 ± 6.85 mm. In the orthoradial sections, women also registered slightly higher values (9.31 ± 6.02 mm) than men (8.25 ± 7.34 mm), although the differences were not statistically significant (*p* = 0.236).

No significant correlation was observed with respect to age (*p* = 0.96) or the length of the perforating implants (*p* = 0.63).

## 4. Discussion

### 4.1. Interpretation

Involvement of the maxillary sinus in the form of odontogenic sinusitis is becoming increasingly common. According to Lupi et al. [16], the most frequent causes are associated with dental procedures such as tooth extractions, endodontic treatments, and implant placement. The substantial demand for implant treatments in the last few decades has made them a first-line option for the rehabilitation of edentulism. However, complications involving the anatomical structures surrounding the implants are increasingly being reported. For this reason, in the posterior maxilla, precise diagnosis and thorough treatment planning are crucial, with prior assessment of the maxillary sinus condition, including its anatomical and pathological features.

Despite the predictability of the interventions performed in close proximity to the maxillary sinus and the high survival rate of both immediate and delayed implants, some studies have reported complications inherent to the invasive nature of these procedures. Although uncommon, such complications can be detected clinically and/or radiologically [17,18]. The most frequently reported complication identified on CBCT scans is SMT.

CBCT-based studies dedicated to the morphometric analysis of head and neck soft tissues are scarce, and even fewer have focused on the Schneiderian membrane and the impact of implants on its morphology.

Based on these observations, the present retrospective study was designed to determine the consequences of sinus floor perforation during dental implant placement on the maxillary sinus membrane, using CBCT analysis and the software NNT Viewer 10.0 for the required measurements. NNT Viewer 10.0 is a highly reliable tool for linear measurements of structures in the dento-maxillofacial region and demonstrates high accuracy in discriminating soft tissues (sinus mucosa) from pneumatic spaces (sinus lumen) [19,20]. The absence of this feature has been a recurring source of error in other software packages [21].

This was verified in the present study by the strong correlation between the linear and area SMT values recorded in the three sections of each patient’s CBCT, supporting both the consistency and accuracy of the measurements and corroborating the actual presence of the condition observed on the scans.

In a systematic review based on 674 CBCT scans, Lechien et al. [22] investigated the various aetiologies of odontogenic sinusitis, in which SMT was the main manifestation. They observed that iatrogenic causes accounted for 65.7% of cases, including treatments of impacted teeth, oroantral communications and implants.

Quirynen et al. [23] examined SMT in 13 CBCT scans of patients undergoing transalveolar sinus floor elevation and subsequent maxillary implant placement, with a 1-month follow-up. One week after sinus floor elevation, the Schneiderian membrane exhibited significant thickening (overall mean: 6.7 mm), which completely resolved 3 weeks later.

Munakata et al. [24] studied the relationship between SMT and various potentially influential factors affecting its dimensions in a sample of 35 patients (11 men and 24 women). They observed that the percentage of thickening greater than 2 mm was 46.2% in men, compared to 11.1% in women. A similar study by Ruhi et al. [25] reported that the morphometric analysis (area and perimeter) of the maxillary sinus mucosa using Auto-CAD 2010 software exhibited significantly higher values in men than in women. These findings contrast with those of the present study, in which women exhibited slightly higher values than men, a difference that might be due to the reduced sample size.

The relationship between age and SMT was also analysed in the study by Munakata et al. [24], which concluded that there was no correlation between age and degree of thickening, consistent with the results of our study. In contrast, Ritter et al. [26] retrospectively analysed 1029 CBCT scans in search of pathological findings in the maxillary sinus and concluded that the prevalence of pathologies in this area was approximately 56.3%. The most frequent finding was SMT, with patients over 60 years of age and men being the most affected.

In this study, the main etiological factor for SMT was the length of the implant portion protruding into the maxillary sinus. We aimed to explore the potential proportional relationship between the extent of this protrusion and SMT. One of the few studies that analysed this relationship was conducted by Jung et al. [27], in which three groups of mongrel dogs received implants protruding into the maxillary sinus at depths of 2 mm, 4 mm and 8 mm. After a 6-month follow-up, they found that the degree of implant penetration into the maxillary sinus did not correlate with the magnitude of thickening observed in any of the groups.

Our findings are consistent with the study mentioned above, confirming the absence of a correlation between the length of the implant protrusion into the sinus and the thickness values measured in the different sections. However, regarding the relationship between the thickening observed and the number of perforating implants placed in the sinus, the sinus membrane density (HU) in the axial section was nearly doubled in areas with two implants compared to those with only one, a statistically significant difference (*p* = 0.029).

In a subsequent clinical trial, Jung et al. [28] assessed the behaviour of the Schneiderian membrane in contact with implants perforating the maxillary sinus floor and protruding more than 4 mm into the sinus cavity, in a sample of nine patients. After a 10-month clinical follow-up, they reported no clinical manifestations of sinusitis. However, CBCT images revealed that 14 of the 23 implants penetrating the sinuses presented SMT around their apices, bounding the sinus floor without involving the ostiomeatal complex. Similarly, Whyte and Boeddinghaus [29] and Tataryn et al. [30], in their respective studies, supported this finding by demonstrating the absence of symptoms in numerous patients, consistent with the results of the present study. In contrast to Jung’s findings, Tabrizi et al. [31], in a clinical trial involving 13 patients with 18 perforating implants, reported that only two patients presented SMT after a 12-month follow-up.

It may be questioned whether the thickening observed in the different sections could be confused with mineral densities. Several CBCT-based studies evaluating membrane thickening density have suggested that increases in sinus membrane density (HU) near perforating implants may be associated with osteogenic processes. Elhamrouni et al. [32] reported this in an animal study in which implants were placed in dog maxillae with sinus floor perforations at three different depths (1, 2 and 3 mm). Five months after the intervention, a histomorphometric analysis of the sacrificed animals revealed ossification around the implants inserted at depths of 1 mm and 2 mm.

The density analysis of SMT in HU in the present study makes it possible to rule out any confusion between the two aforementioned processes, as they can be distinguished by the values obtained. Soft tissues such as the sinus membrane registered values ranging from −700 to 225 HU, whereas calcified tissues measured between 585 and 2850 HU. All HU values of the mucosal thickening in this study fell within the soft tissue range.

Another parameter analysed in this study was the SMT morphological pattern, classified as horizontal or circumferential. In odontogenic sinusitis, a uniform pattern of mucosal thickening is attributed to chronic periodontitis adjacent to the affected teeth. However, in the presence of apical lesions, localised thickening of the Schneiderian membrane may occur [33].

Soikkonen and Ainamo [34] argued that the presence of a horizontal SMT pattern implies the presence of apical irritant stimuli, such as chronic apical periodontitis or deep periodontal pockets. This pattern is usually observed in older individuals and is typically asymptomatic. In contrast, the circumferential SMT pattern is more frequently seen in younger individuals, who have most of their teeth and therefore have a greater potential for irritation-related stimuli. These considerations may not explain the predominance of the circumferential pattern observed in this study, given the mean patient age of 70 years. This raises the question of whether the persistence of chronic sinus mucosa thickening could be interpreted as sinusitis, as suggested by Kato et al. [35] and Insua et al. [36].

### 4.2. Study Limitations

Although the present study was exhaustive and conducted with methodological rigour, certain limitations should be considered. The main limitation lies in the relatively reduced sample size; however, an even greater constraint is the scarcity of studies addressing this topic, which makes comparison of our results challenging. Finally, the one-year follow-up period after implant placement, although longer than in many previous studies, may still be considered short. A longer observation period, including both clinical and radiological assessments, would be more valuable to capture any potential onset of symptomatology [37,38].

## 5. Conclusions

Sinus membrane thickening observed on CBCT scans is often regarded by professionals as a secondary and relatively common finding, given its usually asymptomatic course. Nevertheless, it should be approached as a condition requiring long-term monitoring to ensure the prevention of potential complications. Therefore, it is essential that any treatment planning involving the maxillary sinus includes a preliminary evaluation, taking into account both its anatomical and pathological characteristics. Further studies with an appropriate design—such as prospective or retrospective cohort studies—using standardised implant survival criteria, adequate follow-up, and control of confounding factors are needed to accurately assess the possible clinical consequences and prognosis.

## Figures and Tables

**Table 1:** Inclusion and exclusion criteria for study subjects’ selection.

Inclusion Criteria	Exclusion Criteria
Female or male patients over 18 years old.	Patients restored with implants requiring bone regeneration procedures in the maxillary sinus area.
Patients with no sinus membrane alterations witnessed in the preoperative CBCT.	Patients with rhinosinusitis history.
Patients with implants perforating the maxillary sinus floor.	Patients under treatment with nebulizers or nasal decongestants.
Patients with >2 mm sinus mucosa thickening evaluated by CBCT after the intervention.	

**Table 2:** Patient characteristics and implant perforation measurements.

Patient	Age	Gender	NPI	Affected Side	IPL (mm) Panoramic	IPL (mm) Orthoradial
1	45	M	1	R	0.6	0.6
2	61	F	2	R	6.3	4.1
3	76	M	2	R	2.4	2.1
4	69	M	1	R	1.5	2.1
5	68	M	1	L	4.8	4.5
6	78	F	1	R	1.5	2.1
7	81	F	2	L	0.9	0.6
8	51	F	1	L	2.7	5.1
9	85	F	1	R	2.7	2.1
10	77	F	1	R	2.1	1.5
11	88	F	2	L	2.4	3.3
12	69	M	1	L	0.6	0.3
13	78	F	1	L	1.8	5.1
14	77	F	1	L	2.5	1.2
15	69	M	2	R	2.4	3.3
16	49	M	1	L	1.5	1.2
17	75	F	2	L	5.1	1.2
18	74	M	1	R	4.2	2.4
19	76	M	1	L	1.5	1.5
20	88	F	2	R	1.2	0.9
21	43	M	2	R	2.7	2.2

NPI, number of perforating implants; IPL, implant protrusion length; M, male; F, female; R, right; L, left.

**Table 3:** Sinus mucosa thickening values in terms of pattern, area (mm^2^), density (HU), and maximum height (mm).

Patient	SMT Pattern	SMT Area (mm^2^)			SMT Density (HU)			SMT Max Height (mm) T1-T0	
		Pn	Ort	Ax	Pn	Ort	Ax	Pn	Ort
1	C	221.94	153.36	280.98	242.4	63.4	287.1	17.1	16.7
2	C	160.9	118.26	141.03	74.2	306.8	461.3	11.9	13.1
3	C	117.45	57.15	172.8	261.8	353.9	558.6	3.1	2.1
4	H	201.33	50.4	191.43	119.2	343.3	205.1	11.2	2.7
5	C	348.39	119.07	209.61	165.3	81.2	307.5	19.2	11.7
6	H	321.66	129.42	236.52	86.1	35.8	247.1	11.4	9.2
7	H	114.12	70.74	111.78	152.2	430.8	256.3	17.7	6.0
8	C	289.35	207.9	364.32	196.5	107.7	165.0	19.7	19.5
9	C	506.34	153.09	310.14	166.3	113.2	197.4	18.0	14.9
10	C	116.73	72.75	109.53	145.0	108.3	365.9	15.9	2.3
11	C	411.12	209.88	317.16	128.0	91.4	219.4	21.3	23.6
12	C	169.11	96.39	99.36	23.7	179.5	480.0	16.7	3.6
13	C	112.32	58.95	82.26	96.1	41.8	247.3	2.4	3.0
14	H	199.89	111.33	404.37	21.8	186.6	343.3	9.9	13.2
15	C	234.54	85.5	269.73	71.2	428.5	178.8	9.3	9.8
16	C	78.84	17.55	60.12	366.2	404.8	271.0	4.2	2.8
17	C	219.24	105.39	189.63	179.9	703.4	327.3	8.1	4.5
18	C	173.52	97.74	233.73	248.2	188.7	320.5	5.1	12.4
19	H	136.26	36.63	120.87	72.5	218.4	584.0	3.0	3.2
20	C	223.74	154.71	290.88	335.7	115.5	395.6	3.7	10.5
21	H	238.32	83.97	89.73	295.1	278.1	582.0	4.5	3.4

SMT, sinus mucosa thickening; HU, Hounsfield Units; T1, control scan after one year; T0, initial scan; Pn, panoramic; Ort, orthoradial; Ax, axial; C, circumferential; H, horizontal.

## Data Availability

The original contributions presented in this study are included in the article. Further inquiries can be directed to the corresponding author.

## Abbreviations

The following abbreviations are used in this manuscript:

CBCT	Cone Beam Computed Tomography
SMT	Sinus membrane thickening
HU	Hounsfield units
